# Identification of novel *amrR* deletions as meropenem resistance mechanisms in clinical *Burkholderia pseudomallei* isolates

**DOI:** 10.1128/spectrum.01936-24

**Published:** 2025-03-26

**Authors:** Supichaya Nimnuan-ngam, Shirley Yi Fen Hii, Rathanin Seng, Natnaree Saiprom, Sarunporn Tandhavanant, T. Eoin West, Narisara Chantratita

**Affiliations:** 1 Department of Microbiology and Immunology, Faculty of Tropical Medicine, Mahidol University, Bangkok, Thailand; 2 Bacteriology Unit, Infectious Diseases Research Center, Institute for Medical Research, National Institutes of Health, Ministry of Health Malaysia, Putrajaya, Malaysia; 3 Department of Medical Science, Amnat Charoen Campus, Mahidol University, Amnat Charoen, Thailand; 4 Division of Pulmonary, Critical Care & Sleep Medicine, Harborview Medical Center, University of Washington, Seattle, Washington, USA; 5 Mahidol-Oxford Tropical Medicine Research Unit, Faculty of Tropical Medicine, Mahidol University, Bangkok, Thailand; The University of Tennessee Knoxville, Knoxville, Tennessee, USA

**Keywords:** *amrR *mutation, AmrAB-OprA efflux pump, drug resistance, melioidosis, meropenem, *Burkholderia pseudomallei*

## Abstract

*Burkholderia pseudomallei,* an environmental bacterium, is the causative agent of melioidosis, a potentially fatal infectious disease predominantly found in tropical regions. Despite the bacterium’s intrinsic resistance to numerous antibiotics, the antibiotic resistance mechanisms remain poorly understood. Recently, we identified novel partial deletions in the *amrR* gene of meropenem less-susceptible (MEM-LS) isolates (DR10212A, DR90049A, and DR90031E) obtained from patients with melioidosis. In this study, we performed mutagenesis and quantitative reverse-transcription real-time polymerase chain reaction (RT-qPCR) to validate the roles of these partial deletions in the *amrR* gene in MEM-LS isolates. By introducing wild-type *amrR* fragments from strain K96243 into three parental MEM-LS isolates, we successfully constructed three complemented mutant strains (DR10212A∷K96243-*amrR*, DR90049A∷K96243-*amrR*, and DR90031E∷K96243-*amrR*), which exhibited significantly decreased MEM minimum inhibitory concentrations (MIC) compared with their parental strains. Consistent with the decreased MIC, the expression levels of AmrAB-OprA efflux pump genes (*oprA*, *amrB*, and *amrA*) in the complemented mutant strains were downregulated at least 5-fold compared with the parental isolates, indicating the significant role of the partial *amrR* gene deletions in MEM-LS. Our findings provide more understanding of the MEM resistance mechanisms of clinical isolates of *B. pseudomallei*, thereby enhancing future strategies for the treatment and management of melioidosis.

**IMPORTANCE:**

Antibiotic resistance of *B. pseudomallei* poses a significant threat to patients with melioidosis because it interferes with the recovery process and is associated with high mortality. This study reported that three new mutations involving efflux pumps in *amrR* (H92_S154del, V197del, and A202_R207del) confer resistance to MEM. These mutations were previously detected using whole genome sequencing (WGS) analysis of MEM-LS isolates from melioidosis patients in northeast Thailand. The data from this study provide more understanding of common mechanisms of drug resistance in *B. pseudomallei*. This information is essential for the development of more effective drugs for melioidosis treatment in the future.

## INTRODUCTION

Melioidosis is a life-threatening tropical disease, caused by the environmental bacterium *Burkholderia pseudomallei*. The transmission routes of this bacterium include inoculation, ingestion, and inhalation. The disease is endemic in Southeast Asia and northern Australia and has expanded significantly, encompassing regions across South America, Africa, the Middle East, and southern and eastern Asia (1, 2). The global burden of melioidosis has been estimated to be 165,000 cases and 89,000 deaths annually (1). The treatment of melioidosis is complicated by intrinsic resistance of *B. pseudomallei* to many antibiotics and requires biphasic therapy: at least 10–14 days of intravenous ceftazidime (CAZ) or meropenem (MEM), followed by a 3- to 6-month oral eradication phase using trimethoprim-sulfamethoxazole (SXT) as the preferred agent or, as secondary options, amoxicillin clavulanate (AMC) (3). Although CAZ remains the parental antibiotic of choice in low–middle income countries (LMICs), such as Thailand (2) and Southeast Asian countries (4–6), MEM, with its higher cost, is often reserved for severe cases involving neurological complications or persistent bacteremia (7). Even with treatment using the recommended antibiotics, the mortality rate of melioidosis is unacceptably high, ranging from 25%–40% in Thailand (2, 8, 9).

*B. pseudomallei* is intrinsically resistant to many antibiotics, including penicillin, aminoglycosides, and first- and second-generation cephalosporins (3). The mechanisms of resistance to antibiotics in *B. pseudomallei* include enzymatic inactivation, altered target sites, and efflux from the bacterial cell (10). Although rare, the emergence of antibiotic resistance in *B. pseudomallei* has been reported to be involved in chromosomal alterations, including point mutations and deletions (10). It is believed that changes in the amino acid sequence of PenA, such as from cysteine to tyrosine at position 69 (C69Y), proline to serine at position 167 (P167S), and aspartic acid to glycine at position 240 (D240G), resulting in overexpression of the β-lactamase enzyme and subsequent resistance to CAZ (11–14). In addition to *penA* overexpression, CAZ resistance has also been found to be associated with *penA* gene amplification due to a reversible gene duplication event (12, 15). Certain *B. pseudomallei* strains are also resistant to MEM and SXT, which are likely to be involved in the efflux pump mechanism (16–18). Point mutations of *bepS* and *bepT* (transcriptional regulators) are associated with the upregulation of the BpeEF-OprC efflux pump, which decreases SXT susceptibility (17, 18). Likewise, partial deletions in the *amrR* (repressor) have been found to be associated with the upregulation of the AmrAB-OprA efflux pump and reduced MEM susceptibility (16). AmrAB-OprA belongs to the resistance nodulation division (RND) efflux pump in *B. pseudomallei*, which is implicated in resistance to many classes of antibiotics (19, 20). This pump consists of three components: the membrane fusion protein (MFP), RND transporter, and outer membrane protein (OMP), which are encoded by the genes *amrA* (BPSL1804), *amrB* (BPSL1803), and *oprA* (BPSL1802), respectively (10, 19, 21). The regulator of the *amrAB-oprA* operon is encoded by *amrR* (BPSL1805), which is located upstream of *amrAB-oprA* (11, 22).

We recently analyzed the whole genomes of 1,317 clinical *B. pseudomallei* isolates prospectively collected from patients in Northeast Thailand and identified three meropenem less-susceptible (MEM-LS) isolates (DR10212A, DR90049A, and DR90031E) (12). Compared with the reference strain *B. pseudomallei* K96243, the three strains exhibited novel mutations of the AmrAB-OprA efflux pump: partial *amrR* deletions at V197del (strain DR10212A), A202_R207del (strain DR90049A), and H92_S154del (strain DR90031E) (12). We postulated that these partial *amrR* deletions may play a role in decreasing MEM susceptibility. To the best of our knowledge, the effects of these deletions have not been investigated. In this study, we performed mutagenesis and quantitative reverse transcription polymerase chain reaction (RT-qPCR) on three MEM-LS *B. pseudomallei* strains to elucidate the roles of these partial *amrR* gene deletions in reducing MEM susceptibility. Our results contribute to a better understanding of the molecular mechanisms underlying antimicrobial resistance and provide a foundation for the further development of novel treatment strategies to mitigate the public health burden of melioidosis.

## MATERIALS AND METHODS

### Biosafety approval

This study was approved by the Institutional Biosafety Committee of the Faculty of Tropical Medicine, Mahidol University (MU 2022–031).

### *B. pseudomallei* isolates

The prospective parent clinical study (DORIM) was performed during July 2015 and December 2018 at nine hospitals in northeast Thailand (2). Three of 1,317 clinical isolates collected in this study (DR10212A, DR90049A, and DR90031E) were identified as meropenem-less susceptible as described in Fen SHY et al. (12). The isolates were identified as *B. pseudomallei* by typical colony morphology on Ashdown agar (23), latex agglutination assay (24), RT-PCR using primers and probes specific to TTS1 (25), and whole genome sequencing (12, 26). The primers and probes used are shown in Table 1. All live *B. pseudomallei* isolates were cultured in the biosafety level 3 (BSL-3) laboratory at the Faculty of Tropical Medicine, Mahidol University.

**TABLE 1:** Primers and probes used in this study

Primer and probe	Sequence (5’ – 3’)	Reference
Primer pair and probe for *TTS1* RT-PCR		
BpTT4176F	CGTCTCTATACTGTCGAGCAATCG	(25)
BpTT4290R	CGTGCACACCGGTCAGTATC	(25)
BpTT4208P	CCGGAATCTGGATCACCACCACTTTCC	(25)
Mutagenesis of *amrR*		
amrR12A_F	ATGCTCGACGCGGGCGTCG	This study
amrR12A_R	TCCGCTCCGAACGGCGATT	This study
amrR49A_F	CGTCGCCCGCGCTGCGCG	This study
amrR49A_R	TGCCGCTCGGCCCAACGA	This study
amrR31E_F	GAGATCCTCTACATGAAAT	This study
amrR31E_R	GGTTCTTCGTGTTCAGCGA	This study
Sanger sequencing of *amrR*		
amrR_12–49C_F	ACGCATTCGTAAGGGAGCA	This study
amrR_12–49C_R	GGTGTCCACATCCTTGAAA	This study
amrR_31C_F	ACACGCGCAATTGTTCCTC	This study
amrR_31C_R	CCGATGTCTTCTTCACCGT	This study
Efflux pump expression assay		
16 s rRNA-F	GTGGGGAATTTTGGACAATG	(27)
16 s rRNA-R	CCGGGTATTAGCCAGAATGA	(27)
oprA (BPSL1802) _v2-F	CGAGAAGACGATCCAGACGG	This study
oprA (BPSL1802) _v2-R	TGCTTCAGGCGAATCAGCTC	This study
amrB (BPSL1803) _v2-F	TGTTCGCATGGGTGATCTCCTTG	This study
amrB (BPSL1803) _v2-R	GACCGATTCCTCGACGACCTGC	This study
amrA (BPSL1804) _v2-F	ACGGTGAAGAAGACATCGG	This study
amrA (BPSL1804) _v2-R	CTTGACTTCCTGCCCTTCC	This study

### Determination of antibiotic susceptibility

Broth microdilution (BMD) was performed to determine the minimum inhibitory concentration (MIC) of AMC, CAZ, MEM, and SXT against *B. pseudomallei* isolates, as described previously (12). The antibiotic concentration ranges used for BMD were prepared according to the Clinical & Laboratory Standards Institute (CLSI) guidelines to accurately determine the MICs. The antibiotics and their respective concentration ranges were as follows: CAZ, 0.25–256 µg/mL; MEM, 0.03–32 µg/mL; AMC, 0.06/0.03–64/32 µg/mL; and SXT, 0.03/0.59–32/608 µg/mL (Sigma). Cation-adjusted Mueller Hinton broth containing the 2-fold serial dilutions for the respective antibiotics (as indicated above) in a final volume of 100 µL was added to each well of a microtiter plate. Bacterial suspensions in normal saline were adjusted to a concentration of approximately 5 × 10^5^ CFU/mL. The results were read after 18 h of incubation at 37°C. The MIC breakpoints were as follows: (i) AMC: resistant (R), >32/16 µg/mL; intermediate (I), 16/8 µg/mL; susceptible (S), <8/4 µg/mL; (ii) CAZ: R, >32 µg/mL; I, 16 µg/mL; S, <8 µg/mL; and (iii) SXT: R, >2 µg/mL; S, ≤2 µg/mL (28). There was no MIC breakpoint available for MEM susceptibility in the CLSI guidelines; therefore, EUCAST breakpoints were applied in this case. Isolates were defined as MEM-LS when the MIC exceeded >2 µg/mL (29).

### Construction of *amrR* mutant and complemented strains

*amrR* complemented strains were constructed in strains DR10212A (V197del), DR90049A (A202_R207del), and DR90031E (H92_S154del) using pEXKm5 plasmid replacement, as described previously (12, 27). First, the *amrR* gene sequence of the reference strain, K96243 (K96243-*amrR*), was incorporated in a pUC57 plasmid (Genscript, USA). The design of the sequence included two different restriction sites, flanked by the upstream and downstream sequences of the respective *B. pseudomallei* strains. The pUC57-K96243-*amrR* was transformed into *Escherichia coli* DH5α, followed by overnight incubation at 37°C. This plasmid and pEXKm5 were double digested with NotI and EcoRI (TaKaRa, Japan) for strain DR90031E and with XhoI and EcoRI for strains DR10212A and DR90049A. The correct fragments were excised from the gel and purified using a QIAquick PCR Purification Kit (Qiagen, Germany), followed by ligation into pEXKm5 to create the pEXKm5-K96243-*amrR* insertion fragment using a DNA Ligation Kit, Mighty Mix (TaKaRa, Japan). The plasmid was transformed into *E. coli* RHO3 and cultured on Luria Bertani (LB) agar containing 35 µg/mL kanamycin and 400 µg/mL diaminopimelic acid (DAP). The successfully transformed colonies were confirmed with PCR using the primers listed in Table 1. The selected colony was later introduced into the *B. pseudomallei* (DR10212A, DR90049A, and DR90031E) chromosome by homologous recombination. In brief, the selected colony was counter-selected on tryptone yeast extract agar containing 15% sucrose to obtain the mutants. The *amrR* complemented strains (DR10212A∷K96243-*amrR*, DR90049A∷K96243-*amrR*, and DR90031E∷K96243-*amrR*) were verified by Sanger sequencing (U2Bio, Korea) and whole-genome sequencing. BMD (12) was performed on the parental strains (DR10212A, DR90049A, and DR90031E) and the *amrR* complemented strains to compare the antibiotic susceptibility for AMC, CAZ, MEM, and SXT.

### DNA extraction and Sanger sequencing

The genomic DNA was extracted from 1.5 mL of overnight *B. pseudomallei* culture in LB broth using a QIAamp DNA Mini Kit (Qiagen, Germany), and DNA purification was performed according to the manufacturer’s instructions. The concentration of the DNA samples was determined using a NanoDrop Lite spectrophotometer (Thermo Fisher Scientific, USA), and the quality was assessed by agarose gel electrophoresis. The *amrR* insertion sequences in the complemented strains were verified by Sanger sequencing (U2Bio, Korea) using two primer pairs (Table 1), which were designed using Primer-BLAST (https://www.ncbi.nlm.nih.gov/tools/primer-blast).

### Whole-genome sequencing and analysis

WGS was performed on the three pairs of parental and complemented strains (pair 1, DR10212A and DR10212A∷K96243-*amrR*; pair 2, DR90049A and DR90049A∷K96243-*amrR*; pair 3, DR90031E and DR90031E∷K96243-*amrR*) to confirm the correction of complemented strains. The WGS procedure for the parental isolates (DR10212A, DR90049A, and DR90031E) was previously described by Seng et al. (26). The genomic DNA was extracted from 1.5 mL of overnight *B. pseudomallei* culture in LB broth using a QIAamp DNA Mini Kit (Qiagen, Germany) following the manufacturer’s instruction. The DNA was quantified using a NanoDrop Lite spectrophotometer (Thermo Fisher Scientific, USA), and the quality was assessed by agarose gel electrophoresis. The extracted DNA from the complemented strains was processed for 150-base-read library preparation and subsequently sequenced using an Illumina Hiseq2000 system with 100 cycle runs at U2Bio (Korea). We performed *de novo* assembly of the short-read data using default parameters of Velvet v.1.2.10 (30). The genome completeness and contamination were evaluated using CheckM v.1.2.2 (31). The number of contigs and N50 were assessed by QUAST v.5.0.2 (https://github.com/ablab/quast). The completeness and contamination of all genomes were >99.97% and <0.11%, respectively. The number of contigs and N50 of all genomes were <206 and >72,637, respectively. The genomic variation between the complemented strains and parental isolates was determined by mapping short-read sequences of the complemented strain to the assembled genome of parental isolate using the default parameters of Snippy v.4.6 (https://github.com/tseemann/snippy). The variations were curated using Tablet software (https://ics.hutton.ac.uk/tablet/).

### RNA extraction and transcript quantification

Bacterial culture and RNA extraction were performed in triplicate as described previously (12). Briefly, *B. pseudomallei* isolates were enriched at 37°C in LB broth for 18 h. Then, the bacteria were cultured again in LB broth for 5–7 h or until mid-log phase. RNA was extracted using TRIzol reagent (Invitrogen) and treated with RNase-Free DNase according to the manufacturer’s instructions (Qiagen). The RNA concentration was measured using a NanoDrop Lite spectrophotometer (Thermo Fisher Scientific, USA). Two-step RT-qPCR was performed to evaluate the expression levels of the target genes (*oprA*, *amrB*, and *amrA*). cDNA was prepared using a SuperScript VILO cDNA Synthesis Kit (Invitrogen) with 20 ng/µL of RNA. Quantitative RT-PCR was performed in triplicate in a total volume of 10 µL, containing 5 µL of iTaq Universal SYBR Green Supermix (Bio-Rad), 10 µM of the forward and reverse primers (Table 1)**,** and 1 µL of template. RT-qPCR was carried out in 96-well optical plates using the CFX96 Touch Real-Time PCR Detection System (Bio-Rad, CA, USA). The thermal cycling conditions included initial denaturation for 30 s at 95°C, followed by 40 cycles of 10 s at 95°C and annealing for 30 s at 58°C (*oprA*), 54°C (*amrB*), and 62°C (*amrA*). In each run, a reference gene (16 s rRNA) was used as the internal control, and nuclease-free water was used as non-template control. After amplification, melting curve analysis was conducted by increasing the annealing temperature by 0.1°C stepwise from 65°C to 95°C. The gene expression levels were calculated using the 2^-∆CT^ method, normalized with the reference gene. Data were compared for differences in expression levels between each MEM-LS parental isolate and its corresponding MEM-S mutant isolate using Student’s *t*-test. Differences between groups were considered statistically significant at a *P* value of < 0.05. All quantitative data were expressed as the mean ± standard deviation.

## RESULTS

### Complementation of MEM-LS isolates with wild-type K96243 *amrR* fragment

**Fig 1:**
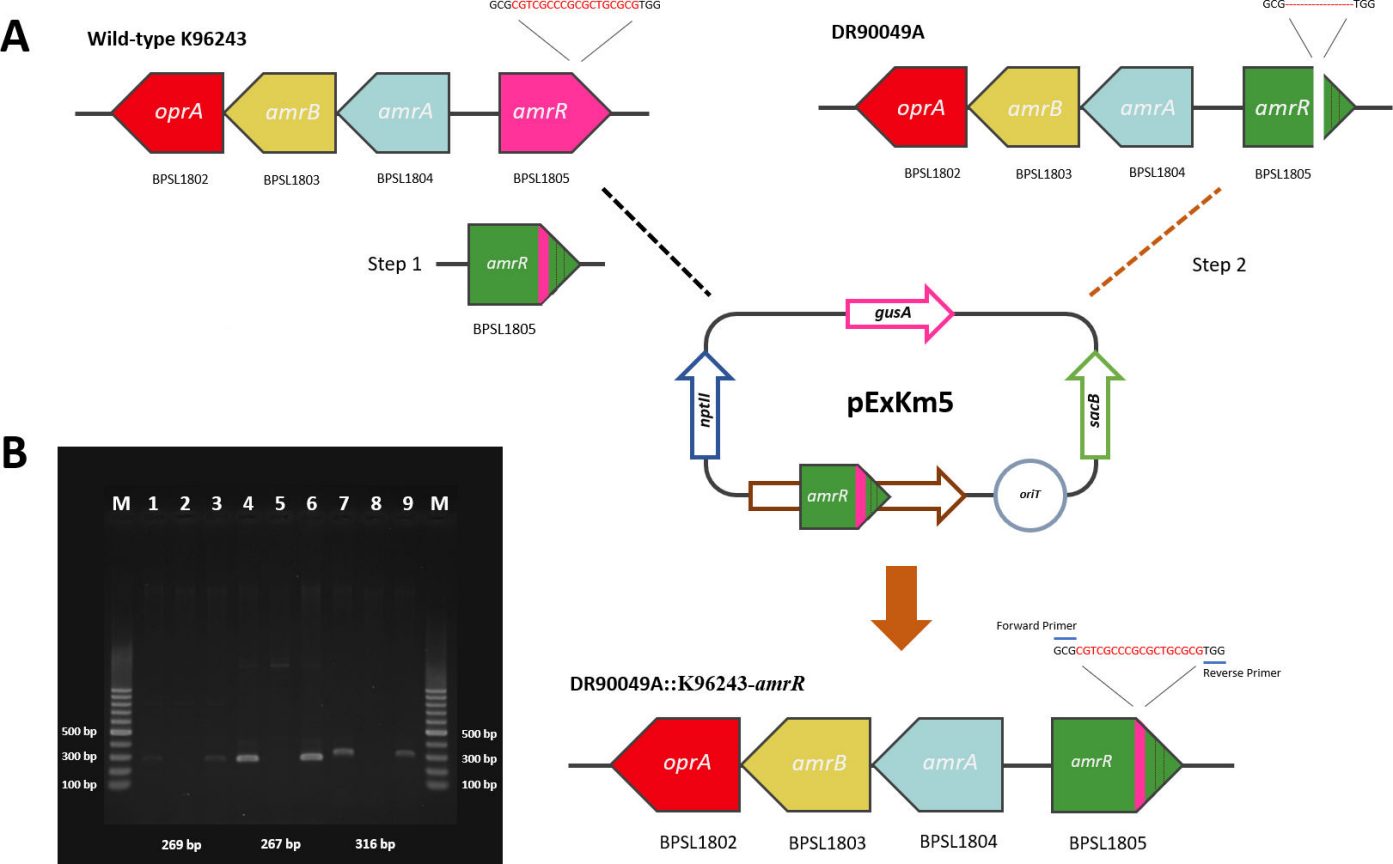
Construction of *amrR* deletion in *B. pseudomallei* strain DR90049A. (**A**) DR90049A∷K96243-*amrR* was created using pExKm5 vector. In step 1, pUC-57 insertion of the *amrR* gene (green) of the AmrAB-OprA efflux pump was synthesized based on wild-type *amrR*-K96243. The pUC-57 containing the desired K96243-*amrR* fragment was transferred to pExKm5 and conjugated to parental strain DR90049A in step 2 to produce DR90049A∷K96243-*amrR*. (**B**) The PCR product of the *amrR* fragment using a specific primer pair from each strain is shown in Table 1. Lanes: M, 100 bp ladder; lanes 1–3, the PCR products of *B. pseudomallei* K96243, DR10212A, and DR10212A∷K96243-*amrR* using amrR12A primers; lanes 4–6, the PCR products of *B. pseudomallei* K96243, DR90049A, and DR90049A∷K96243-*amrR* using amrR49A primers; lanes 7–9, the PCR products of *B. pseudomallei* K96243, DR90031E, and DR90031E∷K96243-*amrR* using amrR31E primers.

**Fig 2:**
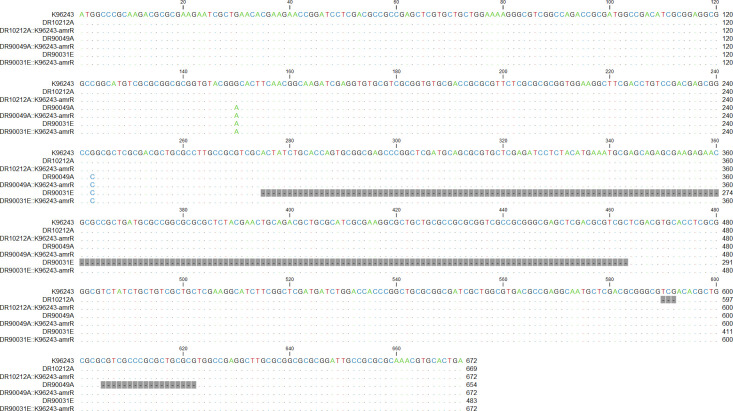
Alignment of the *amrR* sequences in *B. pseudomallei* parental strains (DR10212A, DR90049A, and DR90031E) and wild-type K96243-*amrR* complemented strains (DR10212A∷K96243-*amrR*, DR90049A∷K96243-*amrR*, and DR90031E∷K96243-*amrR*) in comparison to the reference strain K96243. The sequence alignment was performed using MEGA11 software. The deleted regions are highlighted in gray.

To understand the mechanisms of antibiotic resistance in *B. pseudomallei*, we investigated the association between *amrR* deletion mutations and the lowered susceptibility to MEM of three clinical isolates (DR10212A, DR90049A, and DR90031E) identified in our previous study (12). Compared with the genome of *B. pseudomallei* reference strain K96243, the three clinical isolates DR10212A, DR90049A, and DR90031E had different deletion mutations in the *amrR* gene at positions 197 (V197del), 202–207 (A202_R207), and 92–154 (H92_S154del), respectively (12). We postulated that the MEM-LS trait may be associated with deletion mutations in the *amrR* gene of the AmrAB-OprA efflux pump. To test this, first, we performed allelic replacement mutagenesis by complementing the sequences obtained from wild-type K96243 back into the parental strains, producing DR10212A∷K96243-*amrR*, DR90049A∷K96243-*amrR*, and DR90031E∷K96243-*amrR* (Fig. 1A). The PCR products of the *amrR* gene of the complemented strains were observed at 269 bp (for DR10212A∷K96243-*amrR*), 267 bp (for DR90049A∷K96243-*amrR*), and 316 bp (for DR90031E∷K96243-*amrR*), whereas no products were found for the parental strains aligned with the design of our forward primers that were not able to amplify the *amrR* gene with V197del, A202_R207, and H92_S154del deletions (Fig. 1B; Table 1). Sequencing revealed that the *amrR* gene sequence of each complemented strain was identical to that of its parental isolate except for the deletion region, which was replaced with an K96243-*amrR* fragment aligning with our purpose of complemented strain construction (Fig. 2). Furthermore, comparative genomic analysis between the complemented strains and the corresponding parental strains revealed that the K96243-*amrR* fragments were correctly inserted into the *amrR* gene of the complemented strains. In summary, the K96243-*amrR* fragments were successfully inserted into the three MEM-LS isolates, producing complemented mutant strains. These complemented strains were further tested for antibiotic susceptibility and compared with the parental isolates.

### K96243*-amrR* complemented mutant strains are more susceptible to MEM

After introducing K96243-*amrR* fragments into the three MEM-LS strains, we determined the MEM MIC of each complemented strain and compared it with that of the corresponding parental isolate. Compared with DR90049A and DR90031E, the MEM MIC of both DR90049A∷K96243-*amrR* and DR90031E∷K96243-*amrR* was reduced from 4 µg/mL to 1 µg/mL, which were interpreted as MEM-susceptible according to the epidemiological cutoff value (ECOFF) (29). Likewise, the MEM MIC of DR10212A∷K96243-*amrR* was 4-fold lower than that of the parental DR10212A strain (from 16 µg/mL to 4 µg/mL). Although the MEM MIC was reduced after complementation, the DR10212A∷K96243-*amrR* MIC of 4 µg/mL was still categorized as a MEM-LS strain. The persistent less-susceptible phenotype of DR10212A∷K96243-*amrR* raises the possibility that additional factors may also contribute to this MEM-LS. Furthermore, no significant changes were observed in the MICs for AMC, CAZ, and SXT in the complemented isolates (Table 2). Our results suggest that the deletion mutations in the *amrR* regions of DR10212A, DR90049A, and DR90031E are potential causes of the MEM-LS trait.

**TABLE 2:** Antibiotic susceptibility profile of parental *B. pseudomallei* isolates and their corresponding K96243-*amrR* complemented mutant isolates

Strain ID and mutation	MIC (µg/mL) and interpretation			
	CAZ	MEM	AMC	SXT
K96243	2 (S)	1 (S)	4/2 (S)	2/38 (S)
DR10212A (V197del)	128 (**R**)^*a*^	16 (**LS**)	4/2 (S)	4/76 (**R**)
DR10212A∷K96243-*amrR*	128 (**R**)	4 (**LS**)	4/2 (S)	4/76 (**R**)
DR90049A (A202_R207)	2 (S)	4 (**LS**)	4/2 (S)	0.25/4.75 (S)
DR90049A∷K96243-*amrR*	2 (S)	1 (S)	4/2 (S)	0.25/4.75 (S)
DR90031E (H92_S154del)	1 (S)	4 (**LS**)	8/4 (S)	0.25/4.75 (S)
DR90031E∷K96243-*amrR*	1 (S)	1 (S)	8/4 (S)	0.25/4.75 (S)
**CLSI susceptibility breakpoint interpretation (µg/mL)**				
**S**	≤8	NA	≤8/4	≤2/38
**I**	16	NA	16/8	NA
**R**	≥32	NA	≥32/16	≥4/76
**LS**	NA	>2	NA	NA

^*a*^ Bold letters indicate susceptibility test results, where **(R)** represents resistance and **(LS)** denotes less-susceptible isolates of *Burkholderia pseudomallei* to antibiotics.

### Partial deletion of *amrR* enhances the transcription of *amrAB-oprA* efflux pump genes

**Fig 3:**
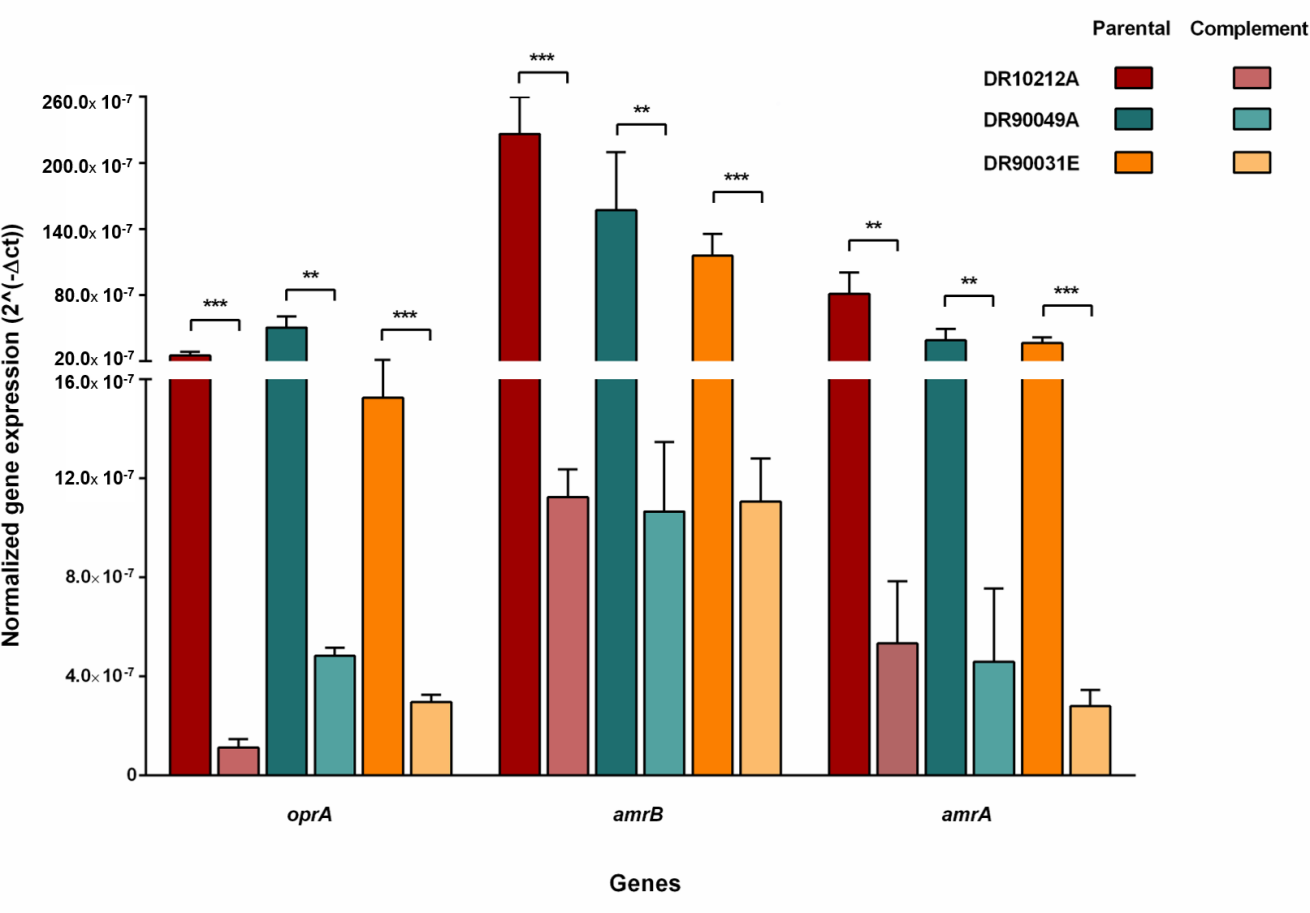
Expression levels of AmrAB-OprA genes in parental isolates and K96243-*amrR* complemented strains. Total RNA was extracted from all tested strains, then reverse-transcribed to cDNA, and the relative levels of *oprA*, *amrB*, and *amrA* were determined by quantitative real-time PCR. The expression experiments were conducted in triplicate, and the expression levels were normalized to 16S rRNA gene. Statistical analysis was performed using Student’s *t*-test: ***, *P* < 0.001; **, *P* < 0.01.

The *amrR* gene encodes a transcriptional regulator protein that controls the expression of the *amrAB-oprA* efflux pump genes, including *oprA, amrB*, and *amrA* (19, 22). We determined the expression levels of these genes in the parental strains (DR10212A, DR90049A, and DR90031E) and their corresponding K96243-*amrR*-complemented isolates (DR10212A∷K96243-*amrR*, DR90049A∷K96243-*amrR*, and DR90031E∷K96243-*amrR*). The results revealed the downregulation of all tested genes in the complemented strains compared with their parental isolates (Fig. 3). For instance, the K96243-*amrR* complemented isolates exhibited a significant reduction in expression of at least 5-fold in the *oprA* gene, 10-fold in the *amrB* gene, and 11-fold in the *amrA* gene compared with the parental strains. The results suggest that the specific mutations within the *amrR* gene (V197del, A202_R207del, and H92_S154del) contribute to the upregulation of *amrAB-oprA* efflux pump transcriptional levels, which are linked to MEM resistance.

## DISCUSSION

*B. pseudomallei* is intrinsically resistant to many antibiotics, posing challenges to the treatment of melioidosis (32). Using WGS analysis, we recently detected partial deletions within three regions of the *amrR* gene, a regulator of AmrAB-OprA efflux pump, in three clinical isolates of MEM-LS *B. pseudomallei* (12). The results from mutagenesis, MIC testing, and RT-qPCR in the current study demonstrated that these partial deletions in the *amrR* gene are associated with the upregulation of *amrAB-oprA* efflux pump genes and MEM resistance in *B. pseudomallei*.

The RND operon, encoding an inner membrane protein (*amrA*) and RND transporter (*amrB*), works with an OMP (*oprA*) to form a tripartite complex under the regulation of *amrR* (19, 33). These proteins work together to actively pump a variety of antimicrobial compounds out of the bacterial cell into the external environment (34–36). In our study, significant changes were observed in the MIC of MEM but not for CAZ, AMC, and SXT upon complementation of two MEM-LS isolates (DR90049A and DR90031E) with wild-type K96243-*amrR*. This indicated that MEM susceptibility is affected by alterations in the *amrR* gene of the AmrAB-OprA efflux pump. However, although the MEM MIC was reduced in DR10212A∷K96243-*amrR* MIC from 16 µg/mL to 4 µg/mL when compared with its parental strain DR10212A, this complemented strain was still categorized as a MEM-LS strain. It is possible that additional factors such as mutations in *penA* may also contribute to this MEM-LS in combination with *amrR* deletions (12).

The alterations in *amrR* include a single-nucleotide polymorphism, and deletion or frameshift mutations were previously reported to confer MEM resistance in Australian isolates (16, 36). These examples were K13fs in MSHR1300, V60_C63del in MSHR6755, G30D in MSHR7929, and A153_D156del in MSHR4083, identified by Sarovich et al. (16), and P81_H223del in MSHR1058, A128_H223del in MSHR8777, and G149fs in MSHR1174, identified by Madden et al. (37). We further analyzed the presence of these MEM-resistance *amrR* mutations in both the parental and complemented strains in our study. We found the absence of these mutations in our strains. Thus, MEM-resistant mutations observed in our study and in Australian isolates are located at the different regions of *amrR* gene. This suggests that mutations in any regions of *amrR* gene might be the potential factors of MEM-resistant.

We recently reported DR10212A as CAZ, SXT, and MEM-resistant strains. The CAZ resistance in this strain was linked to a mutation (P167S) in the *penA* gene (12). Regarding SXT resistance, our study suggests that it may involve genes other than *amrR,* which we have demonstrated in this study to be associated with MEM-LS. The mechanism involved in SXT resistance requires further investigation in future studies.

We evaluated the expression levels of individual genes within the AmrAB-OprA efflux pump and compared them between the parental and complemented mutant isolates. Our findings revealed that all three *amrAB-oprA* efflux pump genes (*oprA*, *amrB*, and *amrA*) were markedly upregulated in parental *B. pseudomallei* isolates compared with the complemented isolates. This supports the regulatory function of AmrR in modulating activity of the AmrAB-OprA efflux pump. The overexpression of *amrB* was also observed in four MEM-LS *B. pseudomallei* strains from Australian patients (MSHR4083, MSHR6755, MSHR9872, and MSHR0052), which was associated with the absence of *amrR* (16, 22). Similar observations have been reported in other bacterial species, such as *Pseudomonas aeruginosa* (38, 39) and *Acinetobacter baumannii* (40), where the upregulation of the MexAB-OprM and AdeABC efflux pumps contributed to decreased meropenem susceptibility.

The evolution of MEM resistance in clinical *B. pseudomallei* isolates remains elusive and may result in therapy failure as MEM is the last resort for melioidosis treatment. The possible reason for emerging antibiotic resistance could be exposure to suboptimal or short-term antibiotic administration during the treatment course (41). The occurrence of MEM-LS strains in clinical isolates could be the result of long-term exposure to antibiotics during antibiotic therapy since two of the three patients experienced recurrent infection. A patient harboring DR90049A had a history of melioidosis 22 months before the enrollment, whereas a patient infected with DR90031E had relapsed for approximately 17 months after the enrollment of our study, and DR90031E strain was collected at the relapse episode (12). Although the MEM susceptibility of the isolate collected at the first episode is not available for patients harboring DR90049A, the first isolate (DR90031A) of patients harboring DR90031E was susceptible to MEM (12), which is different from the second isolate (DR90031E) that was a MEM-LS tested in our study. On our data, the first patient infected with the DR10212A isolate received treatment with MEM and AMC, the second patient infected with DR90049A was treated with ceftriaxone (CTR), doxycycline (DOX), and CAZ during the second episode of infection, while the third patient infected with DR90031E was treated with CTR and AMC at the first episode of infection. AMC, CAZ, CTR, and MEM are all β-lactam antibiotics. Based on the treatment histories, it is possible that exposure to AMC, CAZ, and CTR may cause MEM resistance. Consecutive surveys focusing on clinical isolates of *B. pseudomallei* are essential for tracking the evolution of *B. pseudomallei* and understanding its antibiotic resistance mechanisms.

*B. pseudomallei* is known for its ability to persist and mutate within the human host, contributing to challenges such as relapse infections, which are often associated with enhanced virulence and the development of antibiotic resistance (12, 42, 43). Our study provides insights into the genetic mechanisms of MEM resistance, enhancing our understanding of how the key regulatory gene, *amrR,* contributes to this phenomenon. These findings underscore the importance of long-term surveillance of clinical isolates to track the evolution of *B. pseudomallei* and its resistance mechanisms. Furthermore, the development of effective strategies to prevent relapse infections is essential to minimize resistance-associated mutations and their impact on patient outcomes. This study contributes to the foundation for improved diagnostics, targeted treatments, and public health strategies to combat melioidosis and reduce its burden in endemic regions.

In conclusion, our results suggest that newly identified deletion mutations observed in the *amrR* gene (V197, A202_R207, and H92_S154) present in clinical isolates of *B. pseudomallei* could potentially regulate the overexpression of the AmrAB-OprA efflux pump, leading to reduced MEM susceptibility in *B. pseudomallei*. However, further validation, such as functional assays or interaction studies, is required to fully understand the molecular mechanisms by which these deletions in *amrR* contribute to *amrAB-oprA* efflux expression. Our results provide insights into the antibiotic resistance of *B. pseudomallei,* which is crucial for disease management and further treatment strategies for melioidosis.

## Data Availability

The WGS data for the complemented strains (DR10212A::K96243-*amr*R, DR90049A::K96243-*amr*R, and DR90031E::K96243-*amr*R), and their parental isolates (DR10212A, DR90049A, and DR90031E) were deposited in the DDBJ/ENA/SRA databases and are accessible via NCBI under accession numbers SRR30040063, SRR30040064, SRR30040065, SRR30040066, SRR30040067, and SRR30040068.
